# PINK1-Mediated Phosphorylation of Parkin Boosts Parkin Activity in *Drosophila*

**DOI:** 10.1371/journal.pgen.1004391

**Published:** 2014-06-05

**Authors:** Kahori Shiba-Fukushima, Tsuyoshi Inoshita, Nobutaka Hattori, Yuzuru Imai

**Affiliations:** 1 Department of Neurology, Juntendo University Graduate School of Medicine, Tokyo, Japan; 2 Department of Research for Parkinson's Disease, Juntendo University Graduate School of Medicine, Tokyo, Japan; Stanford University School of Medicine, United States of America

## Abstract

Two genes linked to early onset Parkinson's disease, *PINK1* and *Parkin*, encode a protein kinase and a ubiquitin-ligase, respectively. Both enzymes have been suggested to support mitochondrial quality control. We have reported that Parkin is phosphorylated at Ser65 within the ubiquitin-like domain by PINK1 in mammalian cultured cells. However, it remains unclear whether Parkin phosphorylation is involved in mitochondrial maintenance and activity of dopaminergic neurons *in vivo*. Here, we examined the effects of Parkin phosphorylation in *Drosophila*, in which the phosphorylation residue is conserved at Ser94. Morphological changes of mitochondria caused by the ectopic expression of wild-type Parkin in muscle tissue and brain dopaminergic neurons disappeared in the absence of PINK1. In contrast, phosphomimetic Parkin accelerated mitochondrial fragmentation or aggregation and the degradation of mitochondrial proteins regardless of PINK1 activity, suggesting that the phosphorylation of Parkin boosts its ubiquitin-ligase activity. A non-phosphorylated form of Parkin fully rescued the muscular mitochondrial degeneration due to the loss of PINK1 activity, whereas the introduction of the non-phosphorylated Parkin mutant in *Parkin*-null flies led to the emergence of abnormally fused mitochondria in the muscle tissue. Manipulating the Parkin phosphorylation status affected spontaneous dopamine release in the nerve terminals of dopaminergic neurons, the survivability of dopaminergic neurons and flight activity. Our data reveal that Parkin phosphorylation regulates not only mitochondrial function but also the neuronal activity of dopaminergic neurons *in vivo*, suggesting that the appropriate regulation of Parkin phosphorylation is important for muscular and dopaminergic functions.

## Author Summary

Parkinson's disease is a neurodegenerative disorder caused by degeneration of the midbrain dopaminergic system in addition to other nervous systems. *PINK1* and *parkin*, which encode protein kinase and ubiquitin-ligase, respectively, were identified as the genes responsible for the autosomal recessive form of juvenile Parkinson's disease. These two enzymes are involved in mitochondrial maintenance. Although we previously found that Parkin is phosphorylated by PINK1 in mammalian cultured cells, the physiological significance of this interaction *in vivo* remained unclear. Here, we describe that the phosphorylation of Parkin altered mitochondrial morphology and function in muscle tissue through the degradation of mitochondrial GTPase proteins (such as Mitofusin and Miro) and a mitochondrial respiratory complex I subunit by increasing its ubiquitin-ligase activity. We also found that the dopaminergic expression of both constitutively phosphorylated and non-phosphorylated forms of Parkin affects the flight activity and shortens the lifespan of flies, suggesting that the appropriate phosphorylation of Parkin is important for both dopaminergic activity and the survival of dopaminergic neurons.

## Introduction

Mutations of the *Parkin* and *PINK1* genes cause selective degeneration of midbrain dopaminergic neurons in early-onset Parkinson's disease (PD) [1], [2]. The *Parkin* and *PINK1* genes encode a cytosolic ubiquitin-ligase [3]–[5] and a mitochondrial serine/threonine kinase [6], respectively. Loss of the *Parkin* or *PINK1* genes in *Drosophila* results in degeneration of mitochondria with high energy demands, such as those in muscle and sperm cells [7], [8], and *Drosophila* epistasis analysis has revealed that *PINK1* acts upstream of *Parkin* [9]–[11].

Cell biological studies have demonstrated that Parkin, in cooperation with PINK1, clears damaged mitochondria by utilizing autophagy machinery in a process known as mitophagy [12]–[16]. Reduction of the mitochondrial membrane potential (ΔΨm) leads to accumulation and activation of PINK1 in the mitochondria [15], [17], which recruits Parkin from the cytosol to the mitochondria and activates the ubiquitin-ligase activity of Parkin. Parkin translocates to the mitochondria, where it ubiquitinates and degrades mitochondrial proteins, such as Mitofusin (Mfn) [18], [19] and Miro [20], [21], via the proteasome pathway. These events are thought to reorganize the mitochondrial network and stimulate the recruitment of autophagy machinery. PINK1-dependent recruitment of Parkin to the mitochondria is believed to be the first step of mitophagy [12]–[16]. We have reported that PINK1 phosphorylates Parkin at Ser65 in the ubiquitin-like (Ubl) domain during Parkin translocation, which appears to regulate mitophagy in cultured cells [22]. However, it remains unclear whether Parkin phosphorylation by PINK1 contributes to mitochondrial maintenance and activity of dopaminergic (DA) neurons *in vivo*.

To address this issue, we generated transgenic flies harboring phospho-mutant forms of *Drosophila* Parkin, the phosphorylation site of which is conserved. Transgenic expression of phospho-mutant forms of Parkin in *PINK1* or *Parkin* mutant flies suggests that Parkin phosphorylation by PINK1 enhances the ubiquitin-ligase (E3) activity of Parkin. Our data also provide evidence that overactivation of Parkin by constitutive phosphorylation could lead to tissue dysfunction caused by mitochondrial degeneration whereas absence of Parkin phosphorylation affects DA neuronal activity, leading to the hypothesis that PINK1 is responsible for fine-tuning Parkin activity.

## Results

### *Drosophila* Parkin is phosphorylated in a PINK1-dependent manner

**Figure 1:**
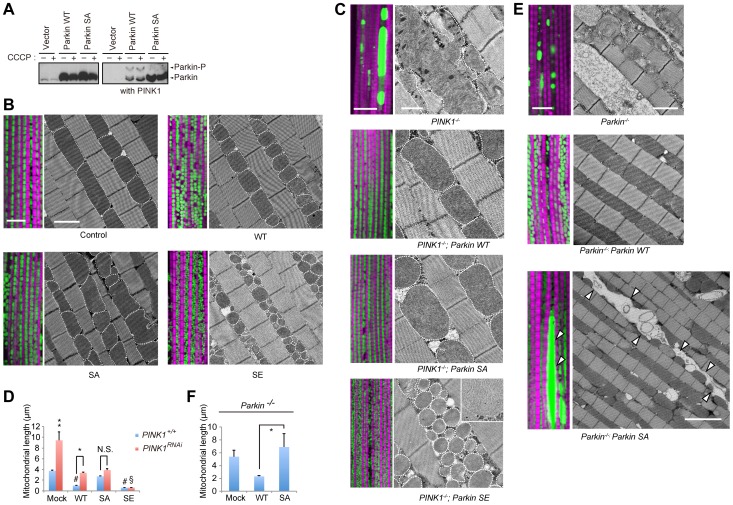
Phosphorylation of the Parkin Ubl domain regulates mitochondrial morphology. (**A**) Parkin is phosphorylated by PINK1 in insect cells. S2 cells transfected with the indicated plasmids with or without *Drosophila* PINK1 were treated with or without 30 µM carbonyl cyanide *m*-chlorophenylhydrazone (CCCP) for 1 h. The cell lysate was subsequently separated on a Phos-tag gel, followed by western blotting with anti-*Drosophila* Parkin. (**B**) The phosphorylation status of Parkin affects the mitochondrial length in muscle tissue. Fluorescent and TEM images of the indirect flight muscle in the indicated genotypes of 14-day-old adult flies are shown. To visualize the mitochondria, the mitoGFP (green) transgene was co-expressed, and the muscle tissue was counterstained with phalloidin (magenta). Mitochondria in the TEM images are outlined with broken lines to highlight their morphology. Scale bars = 10 µm in the fluorescent images and 2 µm in the TEM images. (**C**) Mitochondrial morphology of 14-day-old *PINK1* mutant flies expressing mock, WT Parkin and phospho-mutants. The inset shows a high-magnification TEM image of *PINK1^-/-^; Parkin SE* with intact mitochondrial matrices. Scale bars = 10 µm in the fluorescent images and 2 µm in the TEM images. (**D**) The length of the long axis of the muscle mitochondria was calculated. The data represent the mean ± SE from three flies (*n* = 25 in each). ** *p*<0.01 *vs*. all other genotypes, # *p*<0.01 *vs*. mock with *PINK1^+/+^*, § *p*<0.01 *vs*. *PINK1^RNAi^; WT* or *SA Parkin*, * *p*<0.05, N.S., not significant. (**E**) Mitochondrial morphology of 14-day-old *Parkin* mutant flies expressing mock, WT or SA Parkin. Scale bars = 10 µm in all fluorescent images and 2 µm for *Parkin^-/-^* and *WT Parkin; Parkin^-/-^* and 5 µm for *SA Parkin; Parkin^-/-^* in the TEM images. Arrowheads indicate large mitochondrial aggregates. (**F**) The length of the long axis of the muscle mitochondria was calculated. The data represent the mean ± SE from three flies (*n* = 25 in each). * *p*<0.05. The genotypes are as follows: (**B**) *UAS-mitoGFP/+*; *MHC-GAL4/+* (control), *UAS-mitoGFP/UAS-Parkin*; *MHC-GAL4* (*WT, SA* and *SE Parkin*). (**C**) *PINK1^B9^/Y; UAS-LacZ*; *MHC-GAL4* (*PINK1^-/-^*), *PINK1^B9^/Y; UAS-Parkin*; *MHC-GAL4* (*PINK1^-/-^; WT, SA* or *SE Parkin*). *UAS-mitoGFP; MHC-GAL4, UAS-PINK1 RNAi* crosses were used rather than *PINK1^B9^* crosses for fluorescent images. (**E**) *UAS-mitoGFP/UAS-LacZ*; *Da-GAL4, Parkin^Δ21^/Parkin^1^* (*Parkin^-/-^*), *UAS-mitoGFP/UAS-Parkin*; *Da-GAL4, Parkin^Δ21^/Parkin^1^* (*Parkin^-/-^; WT* or *SA Parkin*).

We and Kondapalli *et al*. have reported that Ser65 in the human Parkin Ubl domain is phosphorylated by PINK1, which is activated by reduction of ΔΨm in cultured cells [22], [23]. The amino acid sequence of the phosphorylation site of *Drosophila* Parkin appears to be conserved [22]. Phos-tag western blotting of *Drosophila* Parkin revealed bands representing PINK1-dependent phosphorylation of Parkin when wild-type (WT) Parkin and *Drosophila* PINK1 were co-transfected into *Drosophila* S2 cells. Introduction of a non-phosphomutated Ser94Ala (SA, corresponding to Ser65Ala in humans) Parkin abolished the phosphorylation bands (Figure 1A, right). The phosphorylation shifts did not occur when ΔΨm was simply disrupted, most likely because of the detection limit of Parkin phosphorylation under this experimental condition (Figure 1A). Next, we generated flies harboring transgenes encoding WT Parkin or non-phospho SA or phospho-mimetic Ser94Glu (SE) mutants. Using the ubiquitous *Da* or eye-specific *GMR* driver of the GAL4-UAS system, we chose at least two independent lines expressing Parkin protein at similar levels in each genotype, and we observed a ∼9-fold increase in Parkin expression relative to endogenous Parkin (data not shown). Because different lines of the same genotype showed similar results, we have presented representative data from each genotype.

### Mutations in the phosphorylation site of Parkin affect mitochondrial morphology

Mitochondria of the indirect flight muscles (IFMs) are prominently affected in flies lacking Parkin [7] or PINK1 activity [9]–[11]. Visualization of mitochondria using mitochondrially targeted GFP (mitoGFP) or transmission electron microscopy analysis revealed that expression of WT Parkin in IFMs shortened the mitochondria in the direction of the long axis compared with a normal control as reported previously [24], and Parkin SE expression resulted in the over-fragmentation of mitochondria (Figure 1B, 1D). In contrast, SA Parkin expression had minor effects on mitochondrial length, although the mitochondrial morphology was less uniform (Figure 1B, 1D).

### SA Parkin does not fully rescue the mitochondrial phenotype caused by loss of endogenous Parkin

The abnormally large, fused mitochondria observed in *PINK1* knockdown or null flies completely disappeared after introduction of WT or SA Parkin (Figure 1C). Intriguingly, the length of the muscular mitochondria, which was reduced by WT Parkin but not SA Parkin expression in the wild-type genetic background, was increased in the *PINK1* knockdown background (Figure 1D). However, mitochondria of *PINK1*-deficient flies expressing SE Parkin were over-fragmented, as observed in flies expressing SE Parkin with endogenous PINK1 activity (i.e., in a wild-type genetic background; Figure 1D). We next examined whether the lack of Parkin phosphorylation alters mitochondrial function when endogenous Parkin activity is removed (Figure 1E). Spotty, large mitochondria labeled with mitoGFP were observed in *Parkin*-deficient flies (Figure 1E, upper), and the wild-type phenotype was recovered by WT Parkin expression (Figure 1E, middle). In contrast, some abnormally fused mitochondria similar to those in *PINK1* mutant flies were observed when SA Parkin was expressed in *Parkin*-deficient flies (Figure 1E, lower). Similarly, SA Parkin failed to suppress the mitochondrial elongation of *Parkin*-deficient flies (Figure 1F).

### Phosphorylation of Parkin increases its E3 activity

**Figure 2:**
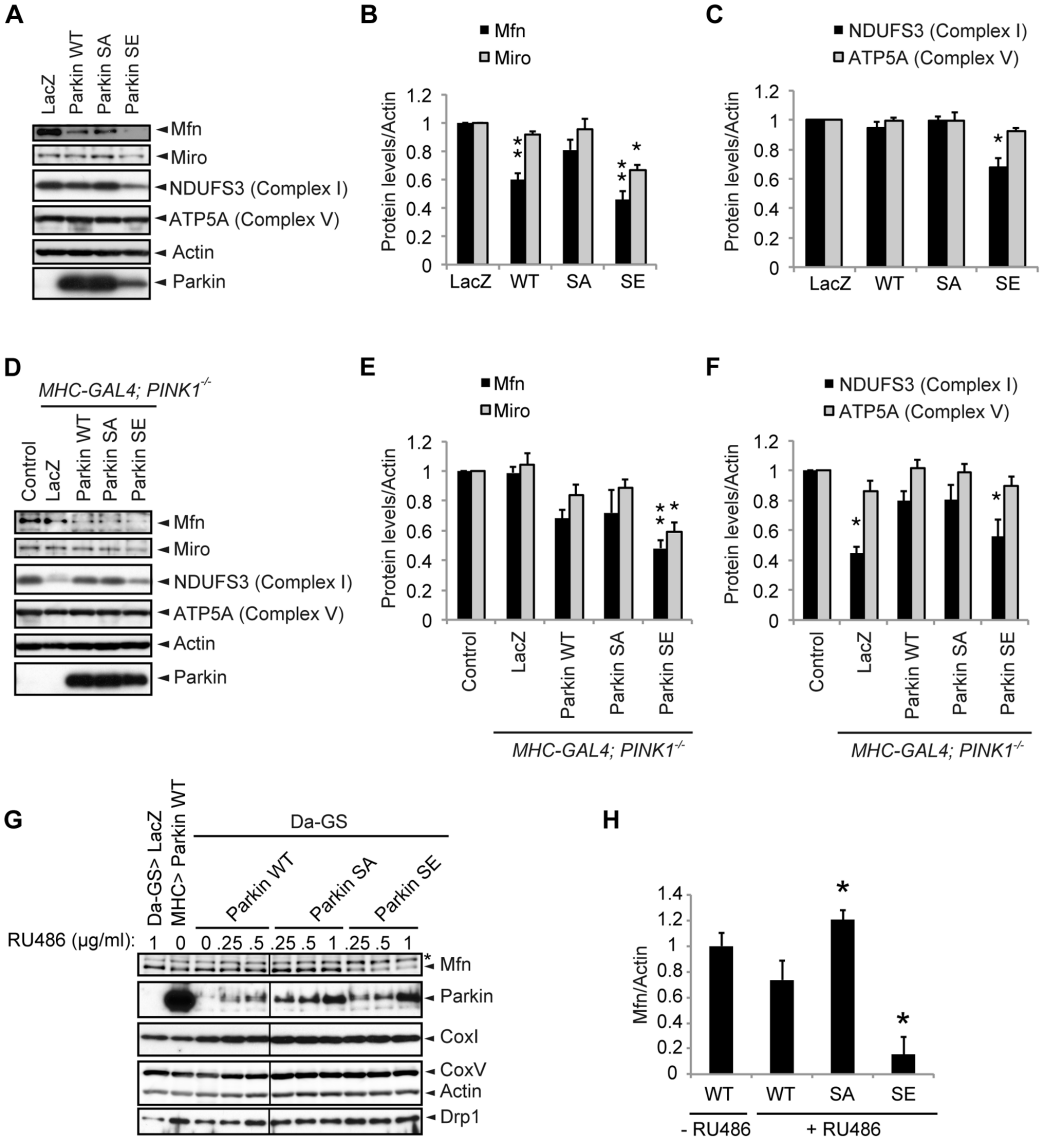
SE Parkin, but not WT or SA, diminishes complex I integrity. (**A**) Parkin (WT, SA or SE) or β-galactosidase (LacZ) was expressed in the thorax muscle using the *MHC* driver. The indicated mitochondrial proteins from the thoraxes of 14-day-old adult flies were analyzed by western blot. Actin was used as a loading control. Note that SE Parkin expression was reduced in this setting (see also Discussion). (**B, C**) The band intensities of the indicated mitochondrial proteins were normalized to each Actin signal. The values (arbitrary units) represent the mean ± SE from five independent samples as in (**A**). ** *p*<0.01, * *p*<0.05 *vs*. *LacZ*. (**D–F**) *LacZ* and *Parkin* were expressed in *PINK1^-/-^* flies using the *MHC* driver as in (**A–C**). *LacZ* expressed in the *w-* background was used as a control (control). (**E**) ** *p*<0.01, * *p*<0.05 *vs. control* or *PINK1^-/-^;LacZ*. (**F**) * *p*<0.05 *vs. control*. (**G, H**) Effects of increasing amounts of Parkin WT, SA and SE on mitochondrial proteins and Drp1. Parkin was induced in adult flies crossed with the ubiquitous *daughterless*–Gene-Switch driver (*Da-GS*) by raising flies with media containing the indicated amounts of RU486 (0, 0.25, 0.5 and 1.0 µg/ml) for 3 days. *UAS-LacZ* crossed with *Da-GS* (*Da-GS>LacZ*) and *Parkin WT* crossed with *MHC-GAL4* (*MHC>Parkin WT*) serve as a negative control and a positive control, respectively. (**H**) Mfn levels were quantified and normalized to each Actin level in flies treated with (+RU486) or without (- RU486) 0.25 µg/ml RU486 as in (**G**). * *p*<0.05 *vs. WT*, +RU486.

Consistent with the results obtained for mitochondrial morphology, as shown in Figure 1, levels of the *Drosophila* mitochondrial outer membrane proteins Mfn and Miro, which regulate mitochondrial morphology and motility and are ubiquitination substrates of Parkin [18], [20], [21], [25], [26], were reduced in SE Parkin-expressing muscles (Figure 2A, 2B). Levels of the mitochondrial complex I subunit NDUFS3 were also reduced in SE Parkin-expressing muscles (Figure 2C). WT, but not SA, Parkin expression reduced Mfn levels to a milder extent than SE Parkin expression. These results suggest that SE Parkin has more potent E3 activity than WT Parkin, whereas SA Parkin has less activity than WT Parkin.

Expression of an unrelated protein, β-galactosidase (LacZ), in *PINK1*-deficient flies failed to rescue the mitochondrial phenotype, and reduction of NDUFS3 levels was observed (Figure 2D, 2F). Introduction of WT or SA Parkin in *PINK1*-deficient flies maintained the NDUFS3 level at the level of the control (Figure 2D, 2F), and the levels of Miro were comparable to those of the control. Mfn levels tended to decrease, although the changes were not statistically significant (Figure 2D, 2E). Expression of SE Parkin in the absence of PINK1 produced results similar to those observed when SE Parkin was expressed in the presence of PINK1 (Figure 2D–F compared with Figure 2A–C). The amounts of Mfn and Miro tended to increase in aged *PINK1*-deficient flies; however, these increases did not reach statistical significance when compared with levels in normal flies expressing β-galactosidase (*PINK1^-/-^*; *LacZ vs*. *control* in Figure S1). The effects of ectopic expression of Parkin in aged *PINK1*-deficient flies were similar to those in young flies (Figure 2 compared with Figure S1) although mild reduction of NDUFS3 levels was observed in *PINK1*-deficient flies expressing SE Parkin with age (Figure S2). Inducible expression of three kinds of Parkin for 3 days after eclosion at various levels also supported the finding that E3 activities of SE Parkin and SA Parkin against Mfn are increased and decreased, respectively (Figure 2G, 2H). In contrast, NDUFS3 levels were not affected in this short duration of Parkin expression (Figure 2G). There was not a good correlation between a mitochondrial fission factor Drp1 level and Parkin E3 activity, suggesting that the morphological change of mitochondria by Parkin is not due to increased expression of Drp1 (Figure 2G). These biochemical results suggest that SA Parkin has lower E3 activity than WT in the presence of endogenous PINK1, whereas SE Parkin has potent E3 activity regardless of PINK1 expression. The results also indicate that an appropriate level of Parkin phosphorylation is required for preservation of mitochondrial complex I and Parkin substrates.

### Constitutive phosphorylation of Parkin compromises mitochondrial function

**Figure 3:**
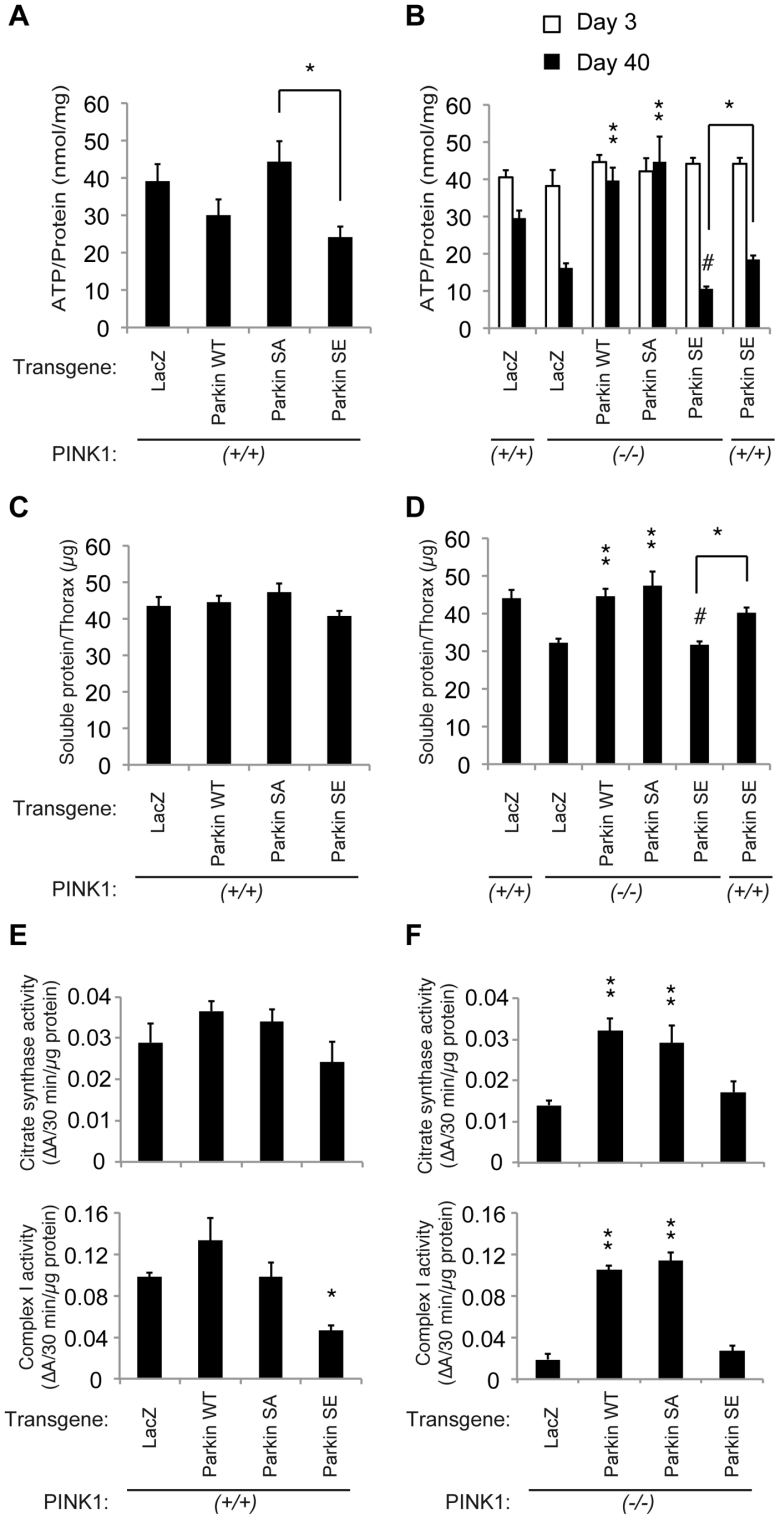
Constitutive expression of SE Parkin compromises mitochondrial function. (**A, B**) ATP contents (**A**, 40-day-old; **B**, 3- or 40-day-old) and (**C, D**) 6 M guanidine-soluble protein levels (40-day-old) of thorax muscle tissues from the indicated genotypes were measured. The transgenes were expressed using the *MHC* driver. ATP contents were normalized against the protein levels. The values represent the mean ± SE from five independent samples. * *p*<0.05, ** *p*<0.01 *vs*. *PINK1^-/-^; LacZ* at the same age. # *p*<0.01 *vs*. *PINK1^+/+^; LacZ, PINK1^-/-^; WT Parkin* and *PINK1^-/-^; SA Parkin* at the same age. (**E, F**) Measurement of mitochondrial enzyme activities. Citrate synthase activity (Upper) and complex I activity (Lower) in the thorax muscle of 40-day-old flies were estimated. (**E**) *MHC-GAL4* crosses in the *PINK1^+/+^* background. (**F**) *MHC-GAL4* crosses in the *PINK1^-/-^* background. * *p*<0.05, ** *p*<0.01 *vs*. *LacZ* (Dunnett test).

Functional disturbance of the mitochondria was estimated by measurement of ATP levels in muscle tissues. Age-dependent reduction of ATP content was observed in muscle tissues expressing SE Parkin (Figure 3A, 3B) and in tissues of *PINK1* null flies (*PINK1^-/-^*; *LacZ*, Figure 3B). The ATP reduction by SE Parkin expression was further exacerbated in the *PINK1* null genetic background (*p*<0.05, *SE Parkin vs. PINK1^-/-^*; *SE Parkin*, Figure 3B). Expression of WT or SA Parkin returned the ATP levels of 40-day-old *PINK1* null flies to normal control levels (not significant, *PINK1^-/-^*; *WT or SA Parkin vs. PINK1^+/+^*; *LacZ*, Figure 3B). We observed that the thorax muscles of *PINK1*-deficient flies (*PINK1^-/-^*; *LacZ*) and SE Parkin flies with or without PINK1 (i.e., *PINK1^+/+^*; *SE Parkin* and *PINK1^-/-^*; *SE Parkin*) became very fragile when we dissected the tissues. Aged SE Parkin flies presented a slight tendency to lose soluble tissue proteins (Figure 3C), and a significant reduction in tissue proteins was detected in aged *PINK1* null flies expressing SE Parkin (Figure 3D). Because PINK1 regulates calcium efflux from the mitochondria via the mitochondrial Na^+^/Ca^2+^ exchanger [27], and is involved in actin dynamics through TORC2-Tricornered kinase pathway [28], Parkin-independent functions of PINK1 might partially contribute to the prevention of ATP shortage and tissue protein loss. Similar results with respect to ATP and protein levels were obtained when we expressed WT and mutant forms of Parkin in *PINK1* knockdown flies, indicating that our *PINK1* knockdown line faithfully recapitulates the *PINK1*-null phenotype (Figure S3). Consistent with the results of ATP production, SE Parkin expression impaired the respiratory complex I activity and failed to rescue the complex I dysfunction by loss of *PINK1* (Figure 3E, F). The activity of citrate synthase, a key enzyme in the Krebs cycle, was reduced in *PINK1* null flies (*p*<0.01, *PINK1^-/-^*; *LacZ vs. PINK1^+/+^*; *LacZ*), which was rescued by WT and SA Parkin, but not SE Parkin (Figure 3E, F).

**Figure 4:**
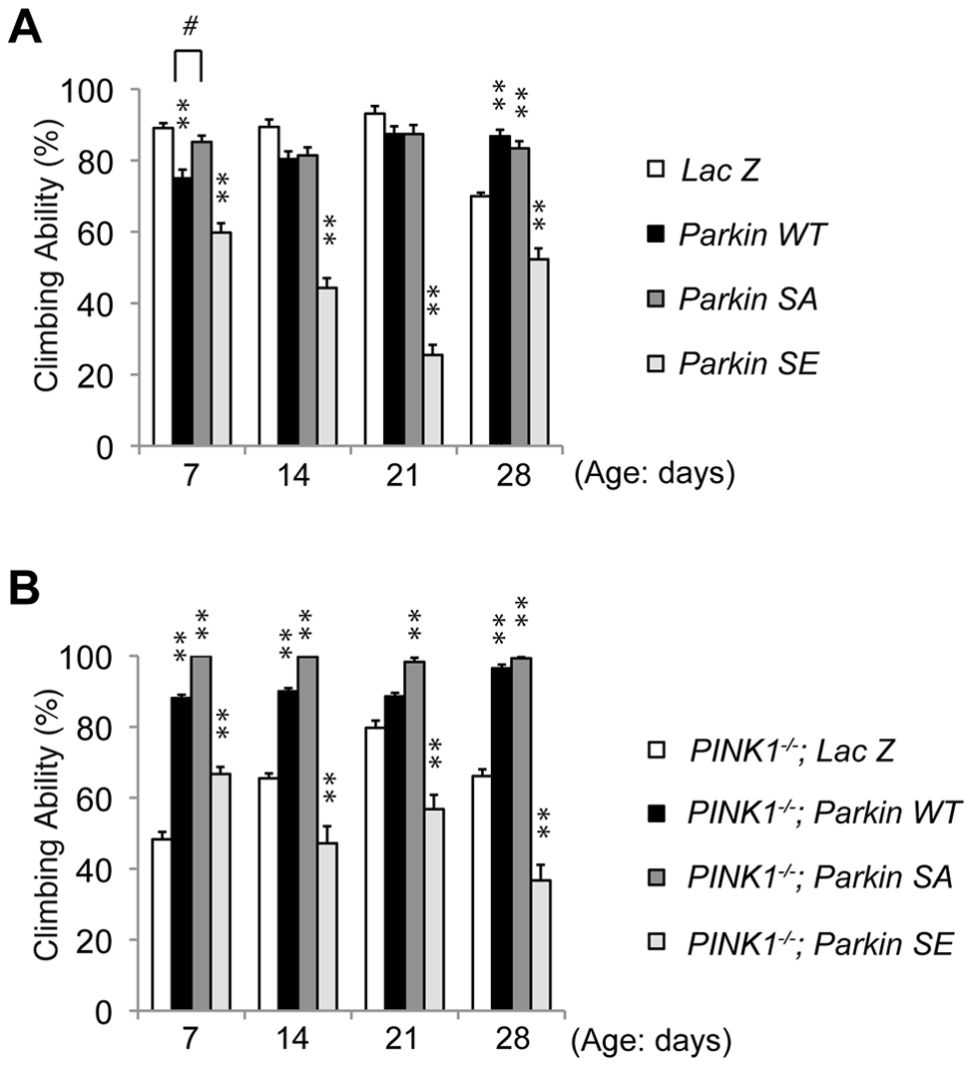
Constitutive expression of SE Parkin impairs motor behavior. (**A**) LacZ and WT, SA or SE Parkin were expressed under the control of *MHC-GAL4* in the *w-* background. WT and SA Parkin improved the motor activity in older flies, whereas SE Parkin compromised motor activity throughout the trials. LacZ served as a control. The values are presented as the mean ± SE from 20 trials. Male flies were used for the assay. **P<0.01 *vs*. *LacZ*; #*P*<0.05. Flies expressing SE Parkin had decreased climbing ability compared with flies expressing WT or SA Parkin throughout the trial (*P*<0.01). (**B**) Muscle-specific expression of Parkin SE under control of *MHC-GAL4* failed to rescue the motor defect of the *PINK1^-/-^* flies. The loss of climbing ability in the *PINK1^-/-^* flies was rescued by expression of WT and SA Parkin. The values represent the mean ± SE from 20 trials. Male flies were used for the assay. **P<0.01 *vs*. *LacZ*. Flies expressing SE Parkin had decreased climbing ability compared with flies expressing WT or SA Parkin throughout the trial (*P*<0.01).

Muscular expression of both WT and SA Parkin improved the age-dependent defect in climbing ability, although WT Parkin expression resulted in a somewhat worse performance than SA in 7-day-old flies (Figure 4A). SE Parkin expression worsened climbing ability compared with a LacZ control. When expressed in the *PINK1* null background, both WT and SA Parkin suppressed the motor defects of *PINK1*-deficient flies (Figure 4B). However, SE Parkin failed to rescue motor behavior after 7-day-old trial (Figure 4B). These data show that constitutive phosphorylation of Parkin is deleterious to mitochondrial function and motor activity.

### SA Parkin functionally rescues mitochondrial degeneration caused by loss of Parkin

Mitochondrial degeneration caused by Parkin loss also caused a reduction in ATP content in thoracic muscle tissues. We ubiquitously expressed WT Parkin and mutant forms of Parkin in *Parkin*-null flies using the *Da* driver. Ubiquitous expression of SE Parkin caused lethality, even in the *Parkin*-null genetic background. Introduction of WT or SA Parkin greatly improved ATP production to a level even higher than that of the normal control (Figure S4A). Similarly, the loss of tissue proteins resulting from Parkin loss was suppressed by expression of WT or SA Parkin (Figure S4B). Mfn and Miro were obviously accumulated in 30-day-old *Parkin* null flies, and the accumulation was prevented by expression of WT or SA Parkin (Figure S4C, S4D). Protein levels of the respiratory complex NDUFS3 were reduced, whereas levels of ATP5A were unchanged in *Parkin*-null flies (Figure S4C, S4E). Mitochondrial Hsp60 was extensively accumulated in *Parkin* null flies, most likely as part of a compensatory mechanism against mitochondrial stress (Figure S4C, S4F) [29]. The alterations in NDUFS3 and Hsp60 levels were also suppressed by both WT and SA Parkin. These results indicate that Parkin phosphorylation is not necessary for mitochondrial function, although an increase in fused mitochondria was observed in *Parkin* null flies expressing SA Parkin (Figure 1F).

### Parkin phosphorylation affects muscular function

**Figure 5:**
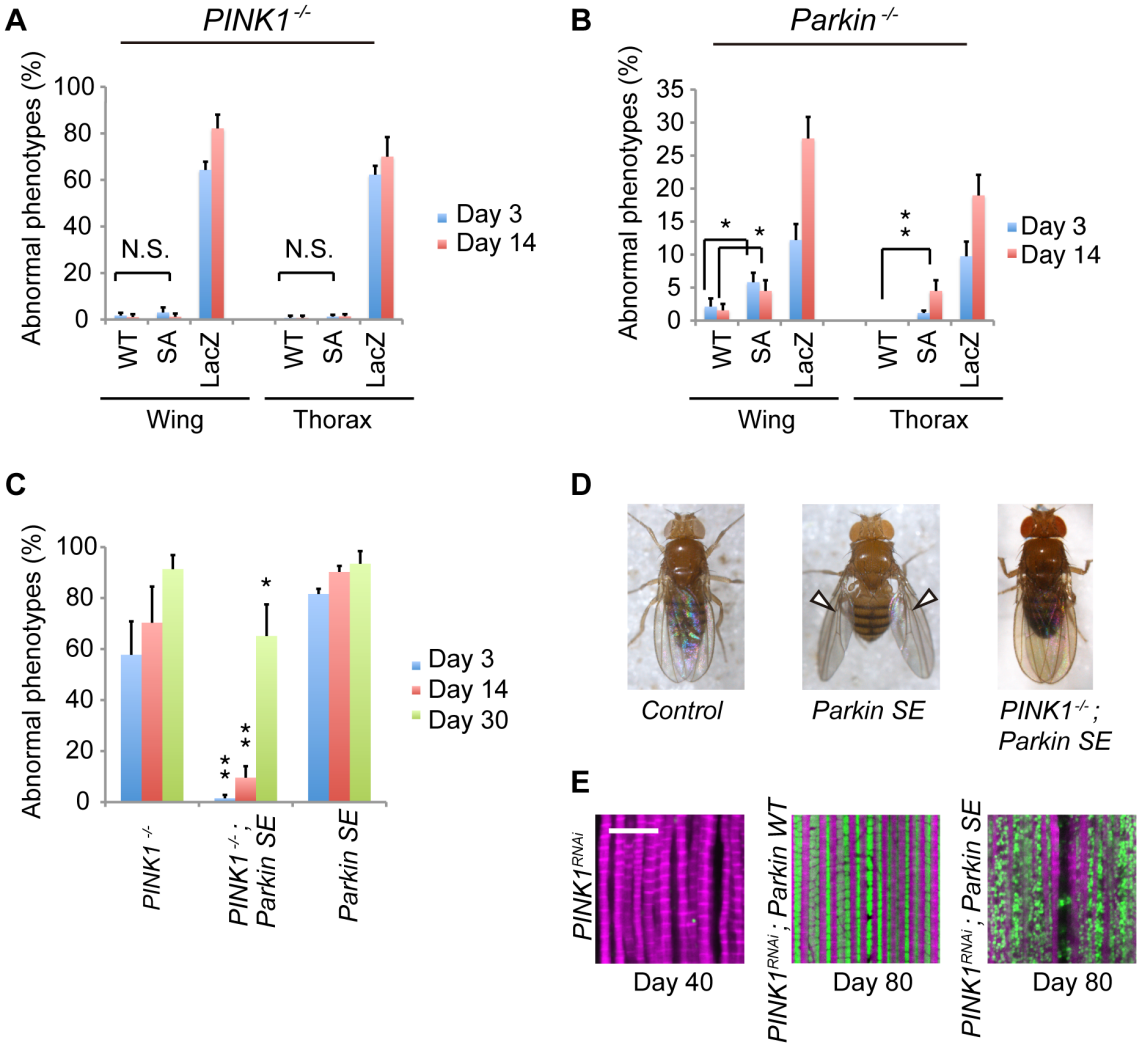
Flies expressing SE Parkin show different wing phenotypes in the presence or absence of PINK1. (**A**) SA and WT Parkin fully rescue the abnormal wing posture and thoracic defect caused by loss of *PINK1* activity (*p*<0.001, *LacZ vs. WT* or *SA*). Graph showing the percentage of flies with abnormal wing posture. Adult male flies aged 3 and 14 days were analyzed. N.S., not significant. *n* = 121–132. (**B**) SA Parkin does not fully rescue the abnormal wing posture and thoracic defect caused by loss of *Parkin* activity. Adult male flies aged 3 and 14 days were analyzed. * *p*<0.05, ** *p*<0.01; *n* = 82–141. (**C**) Expression of SE Parkin in the muscle tissue caused a drooped wing posture, whereas expression of SE Parkin in the *PINK1^-/-^* genetic background improved the abnormal wing posture. The values represent the mean ± SE from four experiments. ** *p*<0.01 *vs*. *PINK1^-/-^* or *SE Parkin* of the same age; * *p*<0.05 *vs*. *PINK1^-/-^* or *SE Parkin* of the same age; *n* = 115–125. (**D**) Representative wing posture of the genotypes indicated in (**C**). Arrowheads indicate drooped wing posture. (**E**) Constitutive expression of SE Parkin preserved the mitoGFP signal throughout the lifespan. *PINK1^RNAi^* and mitoGFP (green) were co-expressed in the muscle tissue together with the indicated transgenes. Scale bar = 10 µm. The genotypes used in (**A, B**) are the same as in Figure 2, and the genotypes in (**C, D**) are *PINK1^B9^/Y; UAS-LacZ*; *MHC-GAL4* (*PINK1^-/-^*), *PINK1^B9^/Y; UAS-Parkin SE*; *MHC-GAL4* (*PINK1^-/-^; Parkin SE*), *+/Y; UAS-Parkin SE*; *MHC-GAL4* (*Parkin SE*).

Abnormal wing postures and dents in the thorax caused by degeneration of mitochondria in IFMs are noticeable phenotypes of flies lacking PINK1 or Parkin activity. Expression of both WT and SA Parkin completely suppressed these phenotypes (Figure 5A). When endogenous Parkin activity was abolished, SA Parkin suppressed the formation of abnormal wing and thorax phenotypes to a lesser extent than WT Parkin (Figure 5B).

Ubiquitous expression of SE Parkin using the *Da* driver caused lethality, and muscle-specific expression of SE Parkin using the *MHC* driver produced an abnormal wing posture similar to that of flies lacking *PINK1* or *Parkin* (Figure 5C, 5D). Although the abnormal wing phenotype was observed even in young adult flies, elimination of PINK1 activity counteracted it, and this effect was weakened by aging (Figure 5C, 5D). Histochemical analyses revealed that expression of SE Parkin in *PINK1*-deficient flies preserved the internal structure of mitochondria (Figure 1D), and the defects in mitochondrial membrane integrity in *PINK1*-knockdown flies appeared to be suppressed by the expression of SE as well as WT Parkin throughout life despite the drooped wing phenotype that occurs with age (Figure 5E).

### Parkin phosphorylation by PINK1 regulates mitochondrial morphology and distribution in DA neurons

**Figure 6:**
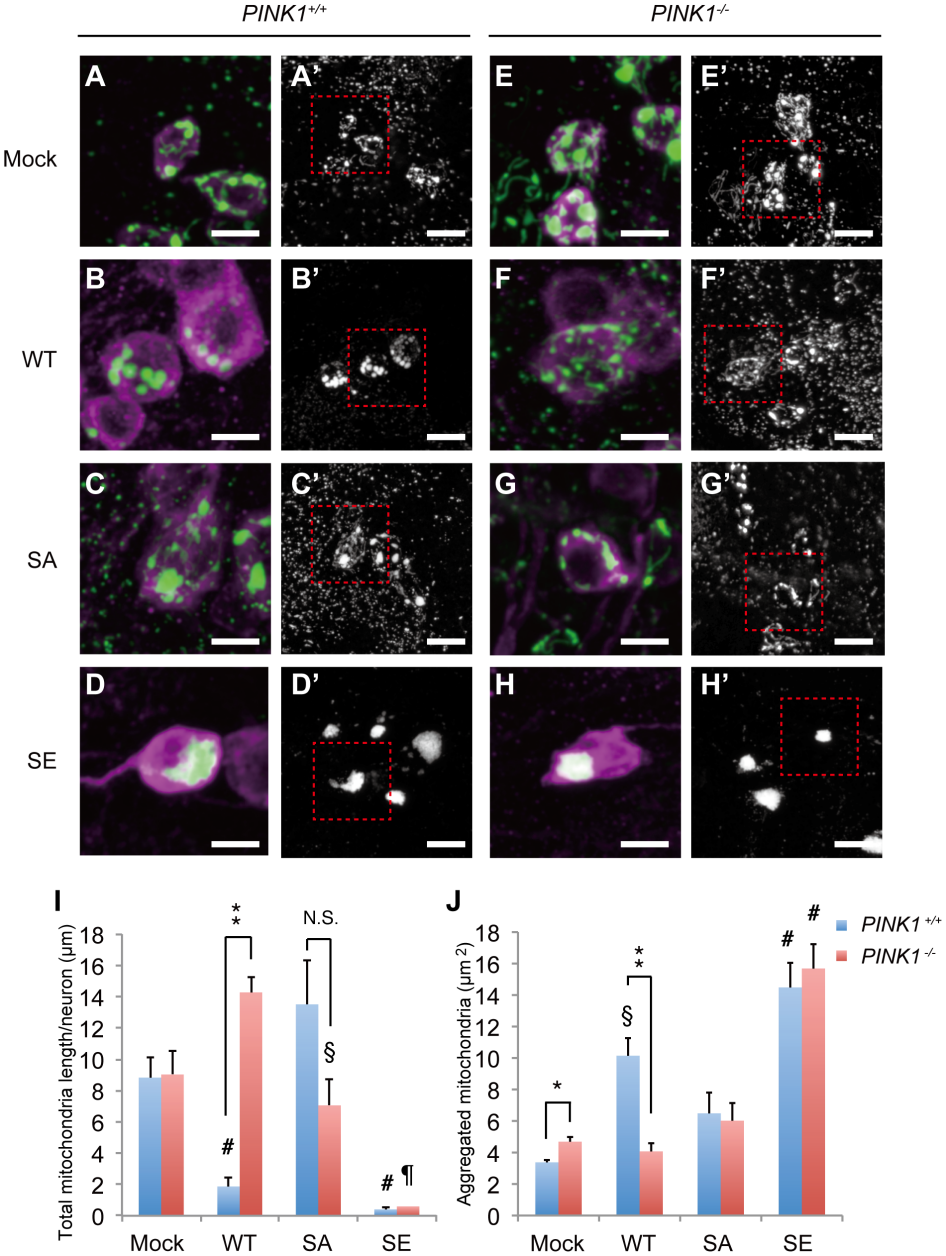
Parkin phosphorylation regulates mitochondrial morphology and the distribution of DA neurons. Mock (**A, A′, E, E′**), WT Parkin (**B, B′, F, F′**), SA Parkin (**C, C′, G, G′**) or SE Parkin (**D, D′, H, H′**) were expressed together with mitoGFP in DA neurons of *PINK1^+/+^* (**A–D′**) or *PINK1^-/-^* (**E–H′**) flies using the *TH* driver. Mitochondria and cell bodies of the PPM1/2 cluster DA neurons were visualized using mitoGFP (green in **A–H** and white in **A′–H′**) and anti-TH staining (magenta in **A–H**), respectively. Images (**A–H**) are higher magnifications of the boxes shown in (**A′–H′**). Scale bars = 5 µm in (**A–H**) and 10 µm in (**A′–H′**). (**i**) Graph showing the total lengths of mitochondria observed in the cell bodies. # *p*<0.05 *vs*. mock in *PINK1^+/+^*, § *p*<0.05 *vs*. WT or SE in *PINK1^-/-^*, ¶ *p*<0.05 *vs*. all other genotypes in *PINK1^-/-^*, ** *p*<0.01, N.S., not significant. (**j**) Graph showing the mean areas of aggregated mitochondria greater than 3 µm in each genotype. # *p*<0.05 *vs*. all other genotypes in *PINK1^+/+^* or *PINK1^-/-^*, § *p*<0.05 *vs*. mock in *PINK1^+/+^*. ** *p*<0.001, * *p*<0.05 (Student's *t*-test).

We next focused on the effects of Parkin phosphorylation in DA neurons, which are affected during PD pathogenesis in humans. Using the DA neuron-specific *TH* driver, WT Parkin and its phosphomutants were expressed in the DA neurons of the adult fly brain. The mitochondrial morphology of the DA neurons in 5-day-old adult flies was analyzed by visualizing mitochondria using mitoGFP. The mitochondria formed tubular networks with several small spherical bodies within the cell bodies of tyrosine hydroxylase (TH)-positive DA neurons of normal control flies (Figure 6A), and many mitochondrial signals were observed outside the cell bodies, which likely represented axonal and dendritic mitochondria transported from the cell bodies of DA neurons (Figure 6A′). Expression of WT Parkin caused a reduction in the number of tubular mitochondria in the cell bodies (Figure 6B), which is consistent with the previous finding that overexpression of PINK1 or Parkin promotes spherical clustering of mitochondria in DA neurons [30], and led to the disappearance of mitochondria outside the cell bodies (Figure 6B′), suggesting that Miro-dependent mitochondrial transport was disturbed. Mitochondrial distribution and morphology in SA Parkin-expressing TH-positive neurons were similar to those in the normal control (Figure 6C, 6C′). Expression of SE Parkin further enhanced the effects of WT Parkin, whereby a single large aggregate of mitochondria appeared in each cell body, and the peripheral mitochondria disappeared (Figure 6D, 6D′).

Given that Parkin is phosphorylated by PINK1, changes in mitochondrial morphology and distribution should be affected in the absence of PINK1. We next examined the mitochondrial morphology of TH-positive neurons in the *PINK1*-null genetic background (Figure 6E-H′), and we compared this morphology with that in the wild-type genetic background (Figure 6A-D′). As previously reported, large aggregates of mitochondria were frequently observed in the cell bodies of flies lacking PINK1 activity (Figure 6E) [10], [30], and mitochondria outside the cell bodies were also observed (Figure 6E′). Introduction of SA and WT Parkin in the absence of PINK1 rescued the phenotype of mitochondrial aggregation and restored the normal mitochondrial morphology (Figure 6F-G′ compared with Figure 6E, 6E′). In TH-positive neurons expressing WT Parkin with or without PINK1, the lack of PINK1 suppressed the mitochondrial aggregation and distribution defects caused by ectopic expression of WT Parkin (Figure 6F, 6F′ compared with Figure 6B, 6B′). In sharp contrast, PINK1 activity did not affect the morphological changes caused by SA Parkin (Figure 6C, 6C′ compared with Figure 6G, 6G′). The effects of SE Parkin expression were similar regardless of PINK1 activity (Figure 6H, 6H′ compared with Figure 6D, 6D′). Taken together, these results indicate that Parkin activity is regulated by PINK1-mediated phosphorylation of the Ubl domain, even under physiological conditions under which PINK1 is not thought to be activated. The total length of tubular mitochondria and the average size of aggregated mitochondria greater than 3 µm^2^ in the cell bodies of TH-positive neurons of each genotype are summarized in Figure 6I and 6J.

### Parkin phosphorylation affects DA neuronal function and lifespan

**Figure 7:**
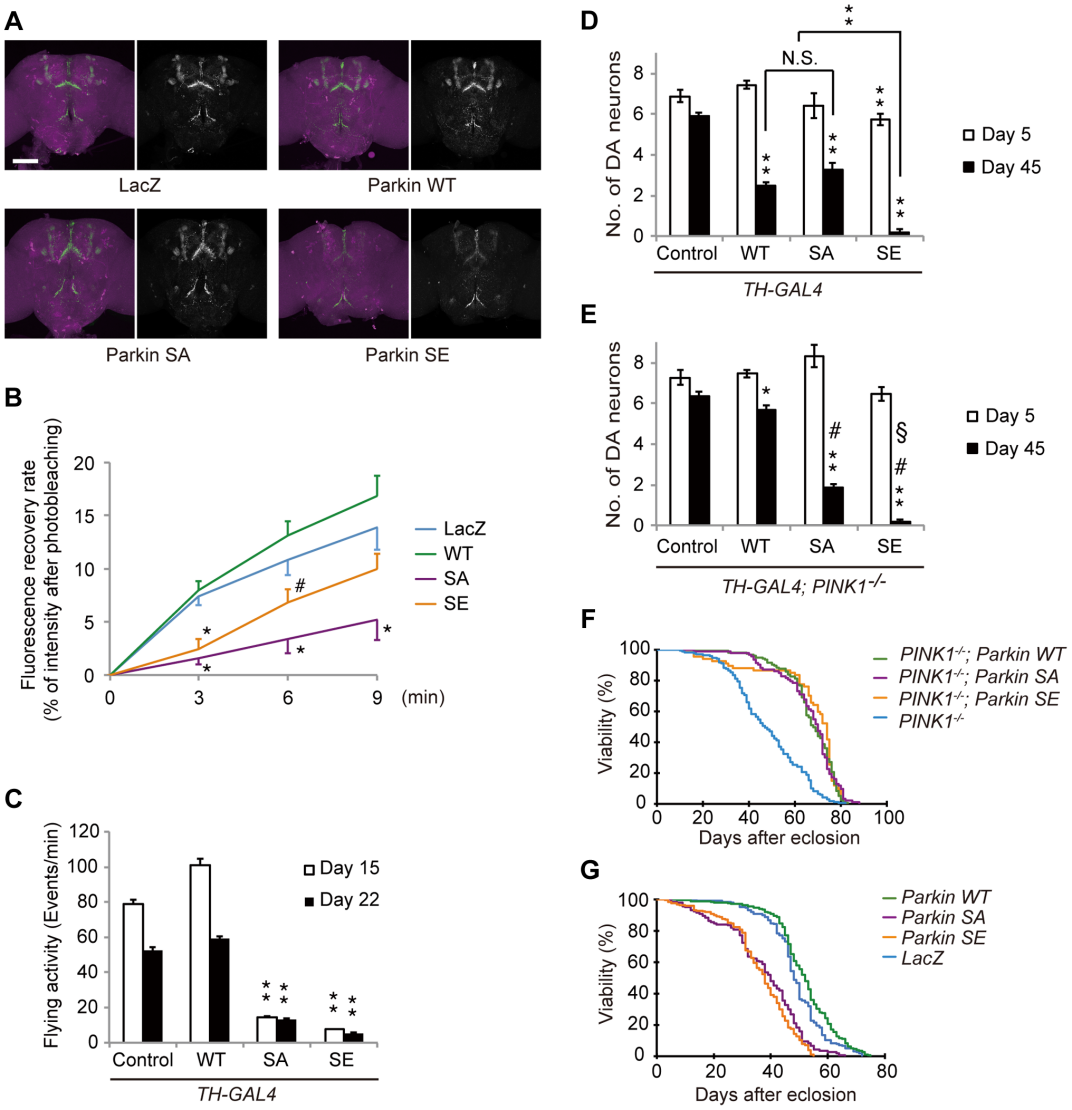
Parkin phosphorylation modulates dopaminergic function and survivability of DA neurons. (**A**) Confocal images of DA neurons (magenta) from the brains of 5-day-old flies expressing VMAT-pHluorin (green) together with the control LacZ and WT, SA or SE Parkin (Left). The *TH* driver was used for transgene expression. VMAT-pHluorin signals are shown as white signals (Right). Scale bar = 100 µm. (**B**) The expression of SA or SE Parkin causes early impairment of vesicular dopamine release in the nerve terminals of DA neurons. The fluorescence recovery rate of DA neuron terminals in cultured neural tissue post-photobleaching was used to estimate the spontaneous DA release rate. Data are shown as the mean ± SE. * *p*<0.05 *vs.* LacZ and WT, # *p*<0.01 *vs*. WT. n = 15–20. (**C**) The flying activity of 15- and 22-day-old males expressing LacZ (control) and those expressing WT, SA and SE Parkin using the *TH* driver. Data are shown as the mean ± SE. ** *p*<0.01 *vs*. control and WT. n = 10. (**D**) Quantification of the number of TH^+^ DA neurons in the PPM1/2 clusters in 5- and 25-day-old males expressing LacZ (control) and those expressing WT, SA and SE Parkin using the *TH* driver. Data are shown as the mean ± SE. ** *p*<0.01 *vs*. control of the same age, # *p*<0.01, N.S., not significant. n = 17–20 for control, WT and SA at 45 days. n = 5–8 for flies at 5 days and for SE flies at 45 days. (**E**) The same assay shown in (**D**) was performed in the *PINK1*-deficient flies. * *p*<0.05, ** *p*<0.01 *vs*. control of the same age, # *p*<0.01 *vs*. control and WT at 45 days, § *p*<0.01 *vs*. SA at 45 days. n = 17–20 at 45 days. n = 6–9 at 5 days. (**F**) Survival curves of flies post-eclosion. Expression of WT, SA and SE Parkin in muscle tissues using the *MHC* driver rescued the shortened lifespan of *PINK1*-deficient flies (*p*<1^−20^ by log-rank test; n = 93–170 male flies); there were no differences in the rescue effect among WT, SA and SE. (**G**) SA Parkin (*P*<1^−11^ by log-rank test; n = 146 male flies) or SE Parkin (*P*<1^−20^; n = 223) expression in DA neurons using the *TH* driver shortened the lifespan compared with the control LacZ (n = 320) and WT Parkin (n = 154).

We next estimated the presynaptic activity of DA neurons expressing mutant forms of Parkin using VMAT-pHluorin, a pH-sensitive form of GFP-conjugated VMAT, to visualize the release of DA [31]. The total expression levels of VMAT-pHluorin were estimated using whole brain samples treated with fixative solution to disrupt the acidic conditions of the synaptic vesicle lumen. In LacZ-expressing DA neurons, VMAT-pHluorin signals were observed in association with DA neuron terminals in the fly brain, including mushroom bodies and the fan-shaped body, as previously reported (Figure 7A) [31]. The localization signals of VMAT-pHluorin in DA neurons expressing WT and SA Parkin were similar to those of LacZ (Figure 7A). In contrast, the VMAT-pHluorin signal was reduced in the SE Parkin-expressing DA neurons (Figure 7A). We next estimated the spontaneous vesicle fusion occurring at the DA neuron terminals using brain tissue cultures expressing VMAT-pHluorin (Figure 7B). Fluorescence recovery after photobleaching (FRAP) was analyzed to estimate the spontaneous neuronal activity. In LacZ and WT Parkin-expressing DA neuron terminals, the fluorescence intensity was recovered to 14–17% of baseline in 9 min. In contrast, the fluorescence recovery in the nerve terminals of DA neurons of SA and SE Parkin flies was reduced compared with that of WT Parkin flies, with only 5% and 10% being recovered, respectively.

The changes in expression of VMAT-pHluorin and dopamine release of the DA nerve terminals in the presence of phospho-mutant forms of Parkin prompted us to test whether PD-associated behaviors could be affected by the status of Parkin phosphorylation. Taking advantage of a startle-induced negative geotaxis, climbing behavior is often investigated in *Drosophila* PD models. However, we found that Parkin expression in DA neurons or throughout the body causes restless behavior, leading the results to not accurately reflect the motor activity. In contrast, flying ability was markedly impaired when SA and SE but not WT Parkin were expressed in DA neurons (Figure 7C and Movie S1), implying that the phosphorylation of Parkin might regulate DA neuronal activity for the motor coordination of flight behavior.

Ectopic expression of WT and SA Parkin using the *TH* driver resulted in an age-dependent loss of DA neurons (Figure 7D). SE Parkin had a more toxic effect; the loss of neurons was detected even in young adult 5-day-old flies (Figure 7D). DA neuronal expression of WT Parkin was less toxic in the absence of PINK1 activity, and SE Parkin expression without PINK1 had a strong neurotoxicity similar to the effect observed with PINK1 (Figure 7E). However, SA Parkin showed more neurotoxicity than did WT Parkin in the absence of PINK1, implying a more complicated molecular mechanism of Parkin regulation in neurons than in muscle tissue (see Discussion).

Finally we investigated whether phospho-mutant forms of Parkin affect lifespan. Loss of *PINK1* shortened the lifespan of *Drosophila*; however, this effect was reversed by muscle-specific expression of WT Parkin. In this setting, both SA and SE Parkin fully restored longevity (Figure 7F). In contrast, DA neuronal expression of both SA and SE Parkin shortened the lifespan compared with that of LacZ and WT Parkin (Figure 7G). These results suggest that the appropriate phosphorylation of Parkin is important for neuronal activity and survival. We summarize our findings using fly models in Table 1.

**Table 1:** Effects of phospho-mutant forms of Parkin on various phenotypes.

Genotypes	Phenotypes					
	Muscle			DA neurons		
	Mitochondrial morphology	Mitochondrial function (ATP)	Climbing ability	Mitochondrial morphology	Neuronal death	Flying ability
Parkin WT	mild fragmentation	normal	improvement^(c)^	mild aggregation	++	normal
Parkin SA	normal	normal	improvement^(c)^	normal	++	impairment
Parkin SE	fragmentation	mild impairment	impairment	aggregation	+++	impairment
PINK1-/-; Parkin WT	normal	improvement^(a)^	improvement^(d)^	normal	+	N.D.
PINK1-/-; Parkin SA	normal	improvement^(a)^	improvement^(d)^	normal	++	N.D.
PINK1-/-; Parkin SE	fragmentation	impairment	impairment^(d)^	aggregation	+++	N.D.
Parkin-/-; Parkin WT	normal	improvement^(b)^	N.D.	N.D.	N.D.	N.D.
Parkin-/-; Parkin SA	partially abnormal	improvement^(b)^	N.D.	N.D.	N.D.	N.D.
Reference data	Fig 1B–F	Fig 3A,B, Figure S4A	Fig 4	Fig 6	Fig 7D,E	Fig 7C

a Compared with *PINK1^-/-^; LacZ*. ^b^Compared with *Parkin^-/-^; mRFP*. ^c^Compared with *LacZ* at 28-day-old. ^d^Compared with *PINK1^-/-^; LacZ* at 28-day-old. N.D., not determined.

## Discussion

We have found that Ser65 in the Ubl domain of human Parkin is phosphorylated by PINK1 upon the reduction of ΔΨm in cultured cells, which appears to be required for mitochondrial translocation of Parkin and degradation of Parkin substrates [22]. Iguchi *et al*. found that phosphorylation of Ser65 is required for ubiquitin-thioester formation with Parkin Cys431, suggesting that this phosphorylation is required for activation of Parkin E3 activity [32]. Consistent with this idea, manipulation of the *Drosophila* Parkin Ser94, which corresponds to the human Parkin Ser65, altered the stability of the known Parkin substrates Mfn and Miro, leading to morphological changes in muscular mitochondria.

We also observed a decrease in the respiratory complex I subunit NDUFS3 upon expression of phospho-mimetic forms of Parkin. A recent *Drosophila* study demonstrated that the PINK1 and Parkin pathway selectively promotes the turnover of respiratory complex proteins [33]. A reduction in the NDUFS3 level was also observed in *PINK1*- or *Parkin*-deficient flies, which indicates that the integrity of respiratory complex I is maintained by a fine balance between PINK1 and Parkin activity. In support of this notion, the ATP production of muscular mitochondria in aged flies expressing SE Parkin was reduced by approximately 40% of a control. Although the motor activity and mitochondrial phenotypes in SE Parkin-expressing flies closely resemble those of *PINK1*- or *Parkin*-deficient flies, a noticeable difference was observed when SE Parkin was expressed in the *PINK1*-deficient genetic background. The drooped-wing phenotype caused by SE Parkin was considerably improved in the absence of PINK1 in young flies. Our previous results indicated that Ser65 in human Parkin is the sole phosphorylation site utilized by PINK1. Similarly, the phos-tag western blot performed in this study also suggests that *Drosophila* Parkin Ser94 is the only phosphorylation site utilized by PINK1. Given that the E3 activity of SE Parkin is equivalent regardless of PINK1 activity, an unknown Parkin regulator(s) is likely modulated by PINK1. Although we cannot rule out the possibility of endogenous Parkin contribution, we prefer this idea, which is supported by our previous finding that Parkin phosphorylation is not sufficient to trigger mitochondrial translocation of Parkin in mammalian cultured cells [22]. Because activated Parkin is preferentially degraded by the proteasome, marked reduction of SE Parkin expression in the wild-type but not *PINK1*-deficient genetic background also suggests that SE Parkin is more active in the presence of PINK1 (Figure 2A, 2D) [22]. However, the abnormal wing phenotype caused by SE Parkin eventually emerged in aged *PINK1*-deficient flies expressing SE Parkin, which suggests that constitutive expression of SE Parkin overwhelmed the function of the PINK1-dependent regulator(s).

Introduction of SA Parkin did not fully restore mitochondrial morphology and the abnormal wing posture in *Parkin*-deficient flies; however, SA Parkin rescued all mitochondrial phenotypes and behavioral defects in *PINK1*-deficient flies. This finding indicates the possibility that endogenous Parkin converts non-phosphorylated latent Parkin into an active form of Parkin, as a study by Lazarou *et al*. demonstrated that Parkin oligomerizes on the mitochondria upon activation [34]. However, co-expression of SA and WT Parkin failed to ensure that ubiquitin was loaded at the catalytic residue of SA Parkin in the cultured cells, suggesting that endogenous Parkin does not activate the E3 activity of SA Parkin *in trans* (Figure S5, lane 6). Thus, a molecular explanation for this observation has yet to be provided.

Expression of Parkin phospho-mutants in the presence or absence of PINK1 activity in DA neurons revealed that Parkin phosphorylation by PINK1 is required for regulation of mitochondrial morphology and motility in DA neurons. Our group and Wang *et al*. have proposed a model in which PINK1 and Parkin prevent damaged mitochondria from moving to the nerve terminals by degrading Miro, an adaptor for microtubule-dependent mitochondrial transport [20], [21]. Supporting this notion, ectopic expression of WT Parkin in DA neurons suppressed mitochondrial distribution outside cell bodies, which was recovered in the *PINK1*-null genetic background. Regardless of the status of PINK1, SE Parkin enhanced the mitochondrial phenotypes observed when WT Parkin was expressed with endogenous PINK1 such that perinuclear accumulation of mitochondria was observed in the cell bodies. These findings indicate that phosphorylation of Parkin by PINK1 boosts its E3 activity, thus regulating mitochondrial motility and morphology through degradation of mitochondrial proteins, such as Miro and Mfn.

We also reveal that Parkin phosphorylation regulates neuronal activity, as our data indicate that spontaneous dopamine release in the nerve terminals and flying activity were compromised in the presence of both SA and SE Parkin expression. The impairment of dopamine release was reported in both *Parkin*-deficient mice [35]–[37] and *PINK1*-deficient mice [38]. The regulation of dopamine release may be independent from the mitochondrial function regulated by PINK1 and Parkin, as Parkin is implicated in the regulation of vesicle trafficking [39]. Our results indicate that both SA and SE Parkin impaired dopamine release, suggesting that the appropriate phosphorylation cycle of Parkin regulates spontaneous dopamine release independently from mitochondrial activity. SE Parkin compromised mitochondrial transport in DA neurons, leading to the perinuclear accumulation of mitochondria. In contrast, reduction of VMAT-pHluorin expression by SE Parkin is unlikely due to inhibition of axonal transport because we did not observe VMAT-pHluorin accumulation in the cell bodies. VMAT might be degraded by activated Parkin.

WT Parkin expression in DA neurons in a wild-type genetic background showed a more toxic effect than those in a *PINK1*-deficient genetic background (Figure 7D, 7E). This difference in neurotoxicity could be explained by the difference in the extent of Parkin phosphorylation by PINK1, leading to a difference in Parkin E3 activity. SA Parkin expression in DA neurons unexpectedly exhibited more neurotoxic activity than did WT Parkin in the *PINK1*-deficent flies (Figure 7E). This result may suggest the existence of a neuron-specific Parkin kinase(s) other than PINK1, although the effect of the kinase on Parkin phosphorylation appears to be smaller than that of PINK1. Tricornered/NDR kinase, which rescues the mitochondrial degeneration caused by the loss of PINK1 in *Drosophila*, could be a candidate of Parkin kinase [28]. Another possibility is that SA Parkin acts in a dominant-negative fashion, as demonstrated by a report that transgenic expression of pathogenic Parkin Q311X resulted in an age-dependent degeneration of DA neurons in the substantia nigra of mice, suggesting that mutant Parkin exerts dominant toxic effects in DA neurons [40].

Inhibition of DA release in the adult brain alters sleep behavior and age-dependent locomotor deficits, which might be associated with PD symptoms. While visual perception is largely maintained in adult flies lacking brain dopamine [41], expression of pathogenic LRRK2, a late–onset PD gene, by the *TH-GAL4* driver resulted in non-autonomous visual neurodegeneration [42]. In another experimental setting, expression of pathogenic LRRK2 by the *TH-GAL4* driver dramatically shortened the lifespan of *Drosophila* [43]. The above reports and our results may suggest that expression of mutant PD gene products including Parkin SA and SE in DA neurons does not only impair DA transmission but also leads to widespread neurodegeneration that affects lifespan non-cell-autonomously.

In summary, we have shown that Parkin phosphorylation by PINK1 drives Parkin E3 activity *in vivo*. Although cell culture studies suggest that PINK1 is inactivated by constitutive breakdown under steady-state conditions, in this study, we have used *Drosophila* models to reveal that endogenous PINK1 precisely controls Parkin activity to maintain the mitochondrial function in muscle tissue and the neuronal function in DA neurons. Our genetic study also suggests the presence of PINK1-regulating factor(s), which may be Parkin regulators. Identification of these unknown factor(s) will be pursued in a further study, and elucidation of the Parkin activation mechanism, including phosphorylation of the Ubl domain, and role of Parkin phosphorylation in neuronal activities will contribute to the identification of a potential therapeutic target in PD pathogenesis.

## Materials and Methods

### *Drosophila* genetics

Fly culture and crosses were performed on standard fly food containing yeast, cornmeal and molasses, and the flies were raised at 25°C. The *w^1118^* (*w^–^*) line was used as a wild-type genetic background. Complementary DNAs for *Drosophila* WT, S94A and S94E Parkin were subcloned into the *pUAST* vector, and *UAS-Parkin WT, S94A and S94E* transgenic lines were generated in the *w^–^* background. All other fly stocks and *GAL4* lines used in this study were obtained from the Bloomington *Drosophila* Stock Center and have been previously described: *UAS-PINK1 RNAi* [11]; *PINK1^B9^* [10]; *park^1^*, *park^Δ21^* [8]; *daughterless*–Gene-Switch [44]; and *VMAT-pHluorin* [31]. *PINK1^B9^* and *park^1^/park^Δ21^* were used as *PINK1-*deficient and *Parkin*-deficient alleles, respectively.

### Antibodies and plasmids

Rabbit anti-*Drosophila* Parkin polyclonal antibody was raised against recombinant MBP-tagged *Drosophila* Parkin (275–482 aa) produced in the *E. coli* strain Rosetta 2 (Novagen). The antibodies used in the western blot analysis were as follows: anti-Parkin (1∶5,000 dilution), anti-Mfn (1∶2,000 dilution; a kind gift of Dr A. Whitworth), anti-Miro (1∶2,000 dilution; a kind gift of Dr E. Zinsmaier), anti-Drp1 (1∶2,000 dilution; a kind gift of Dr L. Pallanck), anti-ATP5A (1∶10,000 dilution; Abcam, 15H4C4), anti-NDUFS3 (1∶10,000 dilution; Abcam, 17D95), anti-Actin (1∶10,000 dilution; Millipore, MAb1501) and anti-Hsp60 (1∶1,000 dilution; Cell Signaling, D307). The antibody used in immunocytochemistry was anti-TH (1∶1,000 dilution; ref. [11]). Complementary DNAs for human and *Drosophila* Parkin and PINK1 were described in previous studies [11], [14], [45]. Parkin phospho-mutants were generated by PCR-based mutagenesis followed by sequence confirmation of the entire gene.

### Western blot analysis

Fly heads and thoraxes were directly homogenized in 20 µl and 40 µl of 3x SDS sample buffer per head and thorax, respectively, using a motor-driven pestle. After centrifugation at 16,000×*g* for 10 min, the supernatants were subjected to western blotting. For Gene-Switch experiments, newly eclosing flies crossed with the *daughterless*–Gene-Switch driver were raised with media containing 5% glucose, 1% agarose and various concentrations of RU486 for 3 days, and their thorax samples were subjected to western blot analysis as described [24]. The band intensity was analyzed using ImageJ software.

### Cell culture

S2 cells were cultured in Schneider's medium (Invitrogen) supplemented with 10% FCS (Invitrogen) and 1% penicillin-streptomycin. The cells were transfected using HilyMax reagent (Dojindo) following the manufacturer's instructions. After 24–48 h, the cells were collected and lysed in lysis buffer containing 0.2% NP-40, 50 mM Tris (pH 7.4), 150 mM NaCl and 10% glycerol supplemented with protease inhibitor (Roche Diagnostics) and phosphatase inhibitor (Pierce) cocktails. Phos-tag western blotting was performed as previously described [46].

### Whole-mount immunostaining and transmission electron microscopy (TEM) analysis

The mitochondrial morphology of the indirect flight muscle and TH-positive neurons was analyzed by whole-mount immunostaining as described previously [46]. The length of the long axis of mitochondria was calculated using ImageJ software. TEM images were obtained at the Laboratory of Ultrastructural Research of Juntendo University.

### VMAT-pHluorin live imaging

Brain tissue samples from 3- to 5-day-old adult flies isolated in HL-3 solution (pH 7.5) [47] were mounted in HL-3 solution for FRAP analysis. Live imaging was performed using a Leica SP5 DM6000 confocal microscope equipped with a 40X oil immersion objective. A 488-nm argon laser applied at 100% power was used to photobleach the whole brain for 2 min. Images were taken as Z-stacks (3 µm slices) at 10% laser power every 3 min. The DA release rates of whole brains were calculated by normalizing against the fluorescent intensity just after photobleaching as the fluorescent recovery rate from 0 to 9 min.

### Lifespan assay, quantification of wing phenotypes, climbing assay and flight assay

For the lifespan studies, approximately 20 adult flies per vial were maintained at 25°C, transferred to fresh fly food and scored for survival every 2 days. To control for isogeny, the driver and *PINK1^B9^* lines were backcrossed to the *w^-^* wild-type background for six generations. All *UAS-Parkin* transgenic flies were generated in the *w^-^* genetic background and thus have matched genetic backgrounds. The number of flies exhibiting defective, abnormal wing posture (held-up or drooped) was determined for each genotype [11]. For flight analysis, 25 control and experimental flies were placed in individual vials (9.3 cm height ×3.5 cm^2^ area), which were then gently tapped to bring the flies down to the vial bottoms. Flight events were counted for one minute at approximately 1 p.m., and the results of ten trials were subjected to averaging. A climbing assay was performed as described previously [48].

### ATP, citrate synthase activity, complex I activity assays and protein measurements

The ATP content in the thorax muscle was measured as described previously with some modifications [48]. Briefly, the thorax of an adult fly was dissected and homogenized in 20 µl of homogenization buffer (6 M guanidine-HCl, 100 mM Tris and 4 mM EDTA [pH 7.8]). After freezing, the samples were centrifuged at 16,000×*g*. The supernatant was diluted 1∶1000 with water for ATP measurement and 1∶10 for measurement of protein concentration. ATP and proteins were measured using the CellTiter-Glo luminescent cell viability assay kit (Promega) and the BCA protein assay kit (Pierce), respectively. Mitochondria isolation, citrate synthase activity assay and complex I activity assay were performed as described previously with some modifications [24]. Briefly, thoraxes were homogenized in mitochondrial isolation medium (250 mM sucrose, 10 mM Tris-HCl, pH 7.5, 0.15 mM MgCl_2_) on ice using a plastic pestle homogenizer, and centrifuged at 500×*g* for 5 min at 4°C. The pellet was resuspended in 50 µl mitochondrial isolation medium per fly. Mitochondrial suspension (5 µl each) was used for citrate synthase activity and complex I activity assays. Complex I activity was calculated as values from which values with 2 µl rotenone were subtracted, normalizing to protein concentrations.

### Statistical analysis

A one-way repeated measures analysis of variance (ANOVA) was used to determine significant differences among multiple groups unless otherwise indicated. If a significant result was achieved (*p*<0.05), the mean values of the control and the specific test group were analyzed using Tukey-Kramer tests.

## Supporting Information

Figure S1 Mitochondrial protein levels in aged *PINK1^-/-^* flies expressing phospho-mutant forms of Parkin. (**A**) Parkin (WT, SA or SE) or β-galactosidase (LacZ) was expressed in the thorax muscle of *PINK1^-/-^* flies using the *MHC* driver as in Figure 2. *LacZ* expressed in the *w*- background was used as a control (control). The indicated mitochondrial proteins from the thoraxes of 40-day-old adult flies were analyzed by western blot. Actin was used as a loading control. (**B**, **C**) The band intensities of the indicated mitochondrial proteins were normalized to each Actin signal. The values (arbitrary units) represent the means ± SE from three independent samples as in (**A**). (**B**) ** *p*<0.01 *vs*. all other genotypes. # *p*<0.01 *vs. control* or *PINK1^-/-^; LacZ*. (**C**) ** *p*<0.01 *vs*. *control*, *PINK1^-/-^; WT Parkin* and *PINK1^-/-^; SA Parkin*. * *p*<0.05 *vs. control*.
(TIF)

Figure S2 Effects of aging on the integrity of the mitochondrial respiratory complexes in *PINK1^+/+^* and *PINK1^-/-^* flies expressing phospho-mutant forms of Parkin. (**A**, **B**) Parkin (WT, SA or SE) or LacZ was expressed in the thorax muscle of *PINK1^+/+^* (**A**) and *PINK1^-/-^* (**B**) flies using the *MHC* driver as in Figure 2. The mitochondrial NDUFS3 and ATP5A from the thoraxes of 3-day-old and 40-day-old adult flies were analyzed by western blot. Actin was used as a loading control. (**C**) The band intensities of NDUFS3 (Cox I) and ATP5A (Cox V) were normalized to each Actin signal. The values (arbitrary units) represent the means ± SE from three independent samples from (**A**, **B**). * *p*<0.05 (Student's *t*-test).
(TIF)

Figure S3 ATP content and protein levels of *PINK1^RNAi^* flies expressing Parkin. ATP content (**A**) and protein levels (**B**) of the thorax muscle in 14-day-old flies were analyzed as in Figure 4. The values represent the means ± SE from five independent samples. ** *p*<0.01 *vs*. LacZ or SE Parkin.
(TIF)

Figure S4 SA Parkin rescues the functional defects in the mitochondrial respiratory complex caused by loss of Parkin. ATP contents (**A**) and protein levels (**B**) of the thorax muscle of 30-day-old flies were analyzed as in Figure 4. The values represent the means ± SE from five independent samples. ** *p*<0.01. (**C–F**) Mitochondrial proteins of the thoraxes of 30-day-old flies were analyzed by western blot as in Figure 3. The values (arbitrary units) represent the means ± SE from three independent samples. (**D**) ** *p*<0.01 *vs*. control or Parkin^-/-^; mRFP, * *p*<0.05 *vs*. Parkin^-/-^; mRFP. (**E**, **F**) ** *p*<0.01 *vs*. all other genotypes.
(TIF)

Figure S5 WT Parkin fails to activate the E3 activity of SA Parkin. HeLa cells were transfected with GFP-tagged human Parkin and untagged human Parkin C431S, in which Ser65 of the Parkin Ubl domain was replaced with Ala (SA) or Glu (SE), or intact (WT) as indicated. The cells were then treated with or without 30 µM CCCP for 3 h. Parkin C431S–ubiquitin oxyester formation (Parkin C431S-Ub) was monitored by western blotting with anti-Parkin. GFP-Parkin-Ub_n_, poly-ubiquitinated GFP-Parkin; asterisk, a processed form of Parkin.
(TIF)

Movie S1 Ectopic expression of SA or SE Parkin in DA neurons causes the defects of flight ability. The genotypes used are: *UAS-LacZ*; *TH-GAL4* (LacZ), *UAS-Parkin WT*; *TH-GAL4* (Parkin WT), *UAS-Parkin SA*; *TH-GAL4* (Parkin SA), *UAS-Parkin SE*; *TH-GAL4* (Parkin SE).
(M4V)
