# Supplementary figures and images for: PINK1-Mediated Phosphorylation of Parkin Boosts Parkin Activity in Drosophila
Source: PLoS Genet. 2014 Jun 5;10(6):e1004391. doi: 10.1371/journal.pgen.1004391 (PMC4046931; doi:10.1371/journal.pgen.1004391)

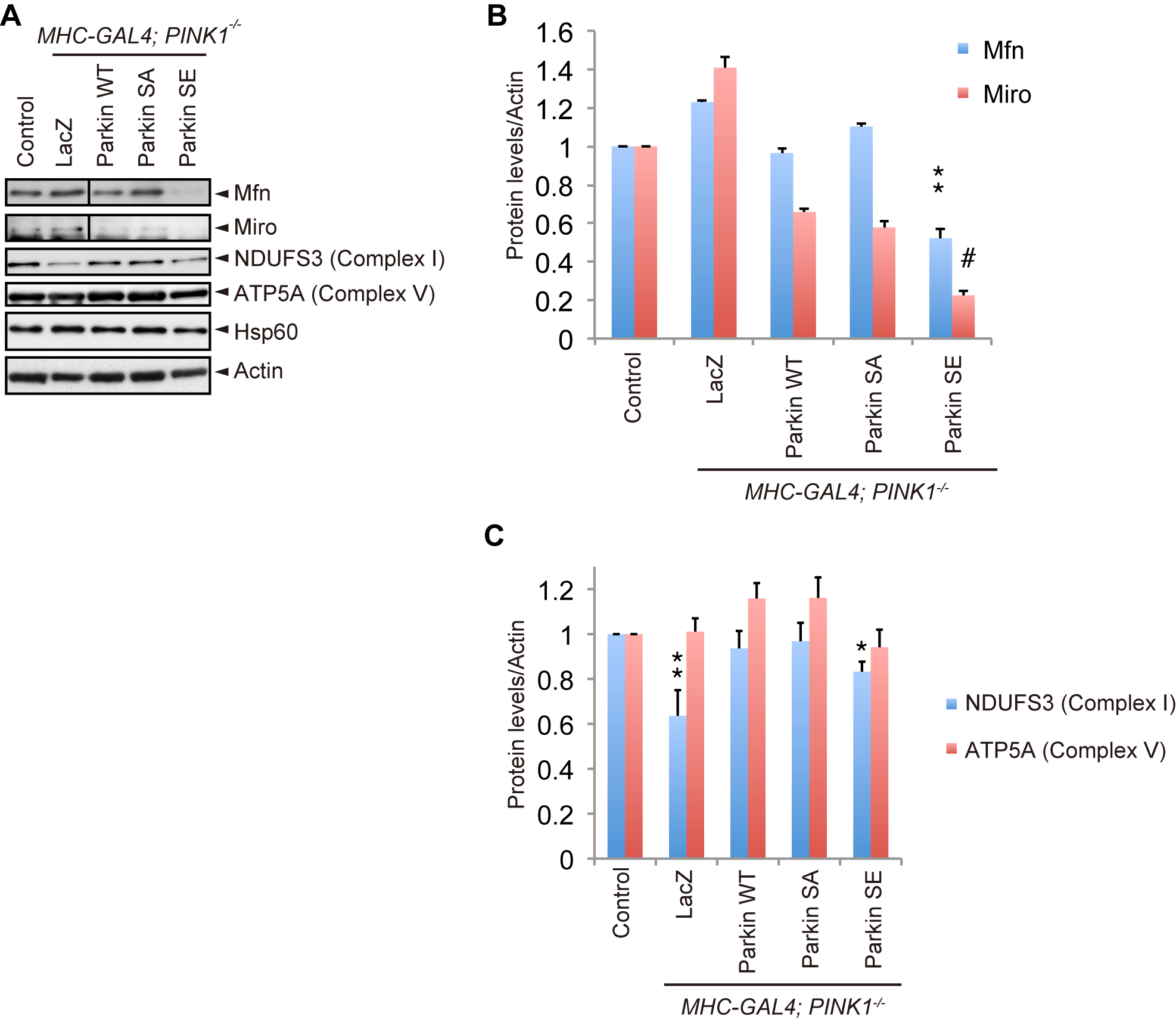

Supplement: Figure S1 — Mitochondrial protein levels in aged PINK1-/- flies expressing phospho-mutant forms of Parkin. (A) Parkin (WT, SA or SE) or β-galactosidase (LacZ) was expressed in the thorax muscle of PINK1-/- flies using the MHC driver as in Figure 2. LacZ expressed in the w- background was used as a control (control). The indicated mitochondrial proteins from the thoraxes of 40-day-old adult flies were analyzed by western blot. Actin was used as a loading control. (B, C) The band intensities of the indicated mitochondrial proteins were normalized to each Actin signal. The values (arbitrary units) represent the means ± SE from three independent samples as in (A). (B) ** p<0.01 vs. all other genotypes. # p<0.01 vs. control or PINK1-/-; LacZ. (C) ** p<0.01 vs. control, PINK1-/-; WT Parkin and PINK1-/-; SA Parkin. * p<0.05 vs. control. (TIF) [file pgen.1004391.s001.tif]

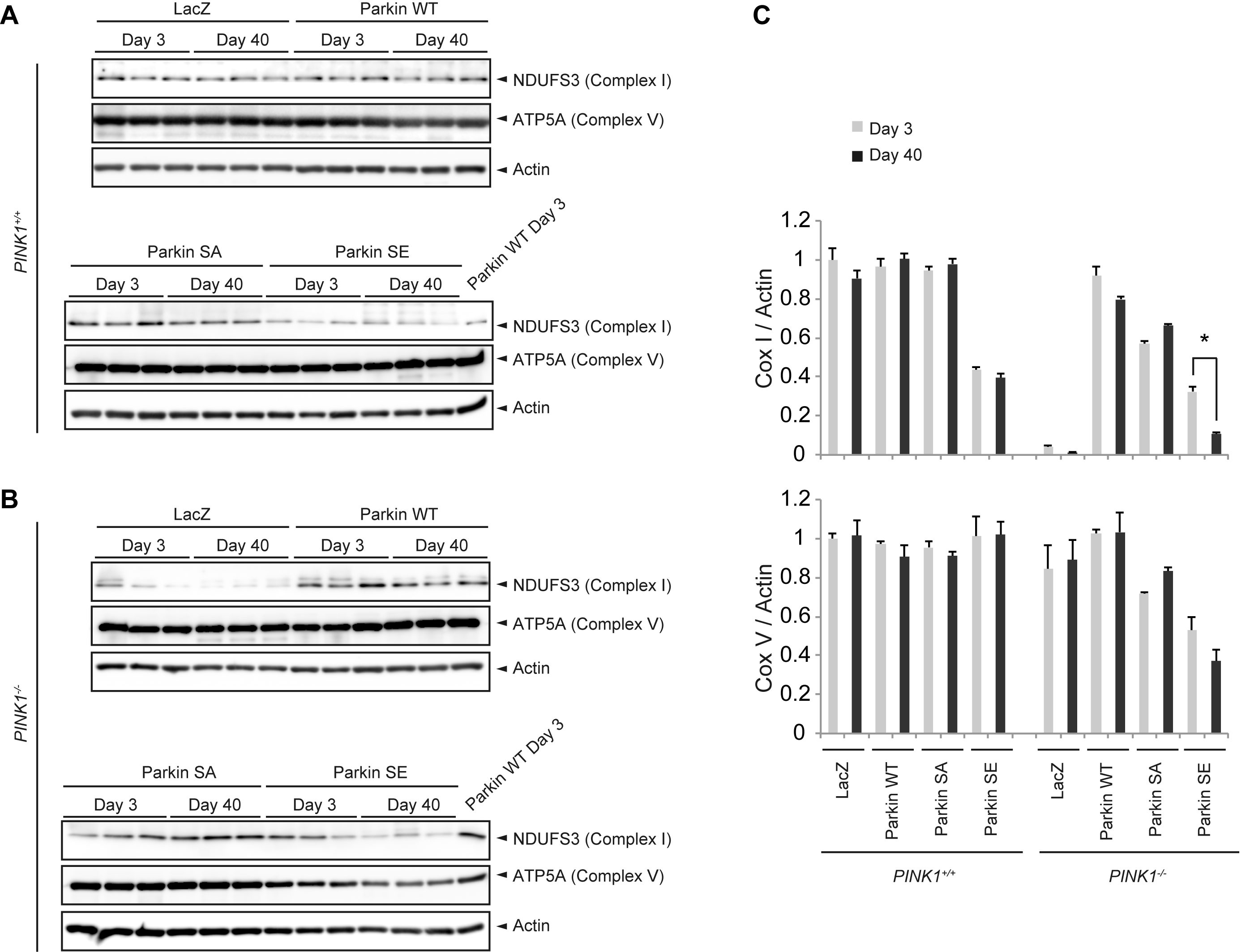

Supplement: Figure S2 — Effects of aging on the integrity of the mitochondrial respiratory complexes in PINK1+/+ and PINK1-/- flies expressing phospho-mutant forms of Parkin. (A, B) Parkin (WT, SA or SE) or LacZ was expressed in the thorax muscle of PINK1+/+ (A) and PINK1-/- (B) flies using the MHC driver as in Figure 2. The mitochondrial NDUFS3 and ATP5A from the thoraxes of 3-day-old and 40-day-old adult flies were analyzed by western blot. Actin was used as a loading control. (C) The band intensities of NDUFS3 (Cox I) and ATP5A (Cox V) were normalized to each Actin signal. The values (arbitrary units) represent the means ± SE from three independent samples from (A, B). * p<0.05 (Student's t-test). (TIF) [file pgen.1004391.s002.tif]

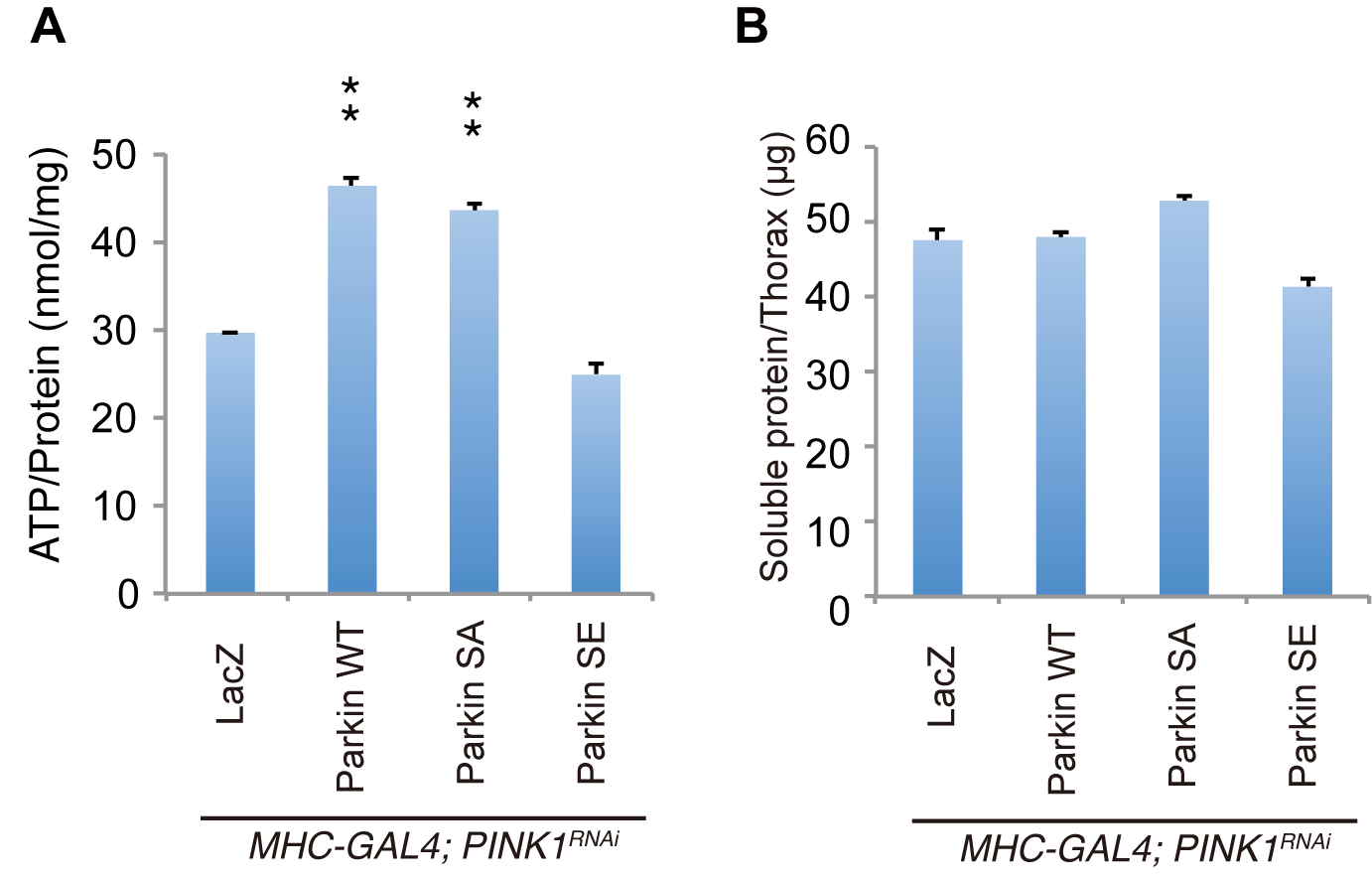

Supplement: Figure S3 — ATP content and protein levels of PINK1RNAi flies expressing Parkin. ATP content (A) and protein levels (B) of the thorax muscle in 14-day-old flies were analyzed as in Figure 4. The values represent the means ± SE from five independent samples. ** p<0.01 vs. LacZ or SE Parkin. (TIF) [file pgen.1004391.s003.tif]

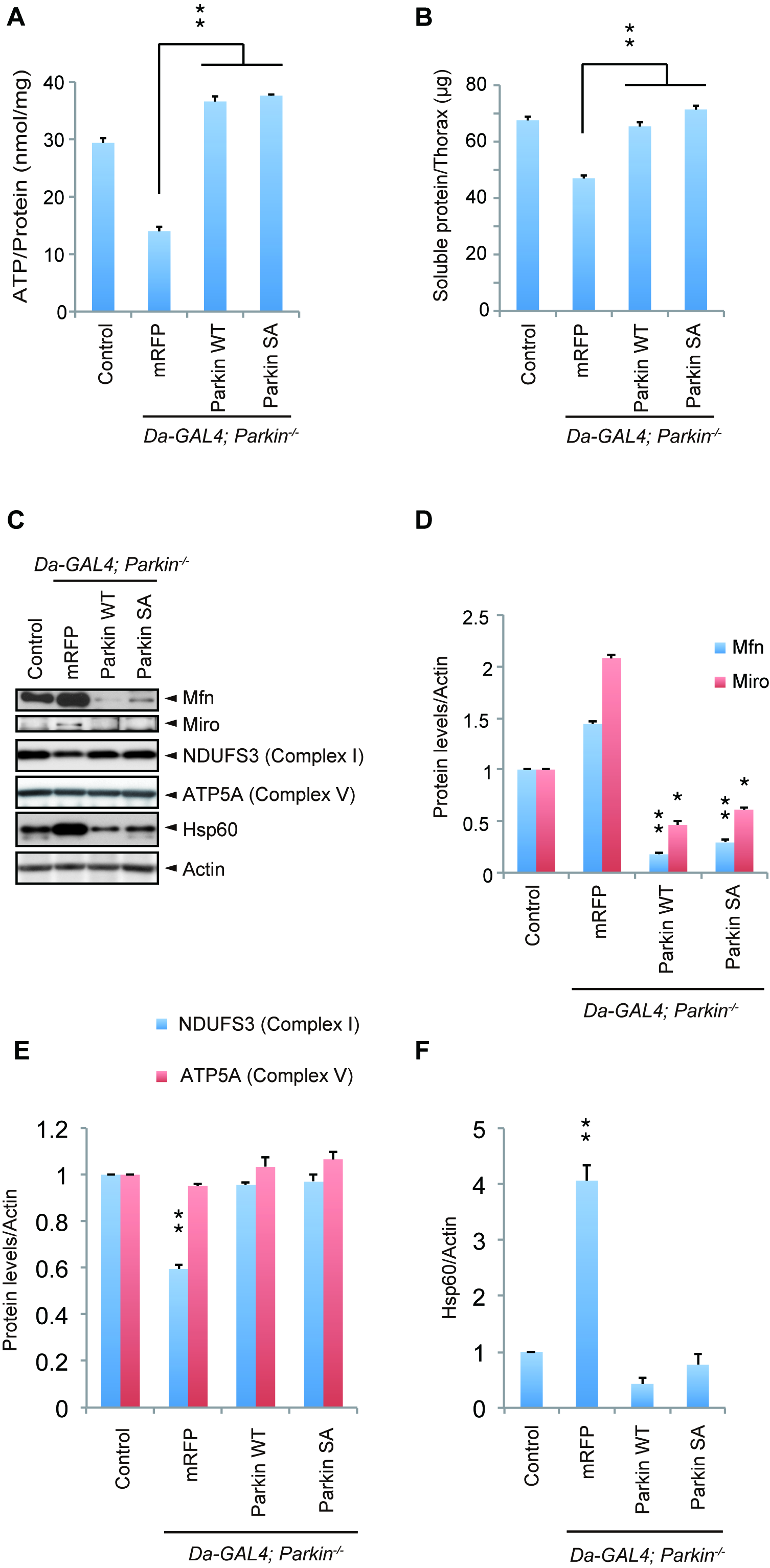

Supplement: Figure S4 — SA Parkin rescues the functional defects in the mitochondrial respiratory complex caused by loss of Parkin. ATP contents (A) and protein levels (B) of the thorax muscle of 30-day-old flies were analyzed as in Figure 4. The values represent the means ± SE from five independent samples. ** p<0.01. (C–F) Mitochondrial proteins of the thoraxes of 30-day-old flies were analyzed by western blot as in Figure 3. The values (arbitrary units) represent the means ± SE from three independent samples. (D) ** p<0.01 vs. control or Parkin-/-; mRFP, * p<0.05 vs. Parkin-/-; mRFP. (E, F) ** p<0.01 vs. all other genotypes. (TIF) [file pgen.1004391.s004.tif]

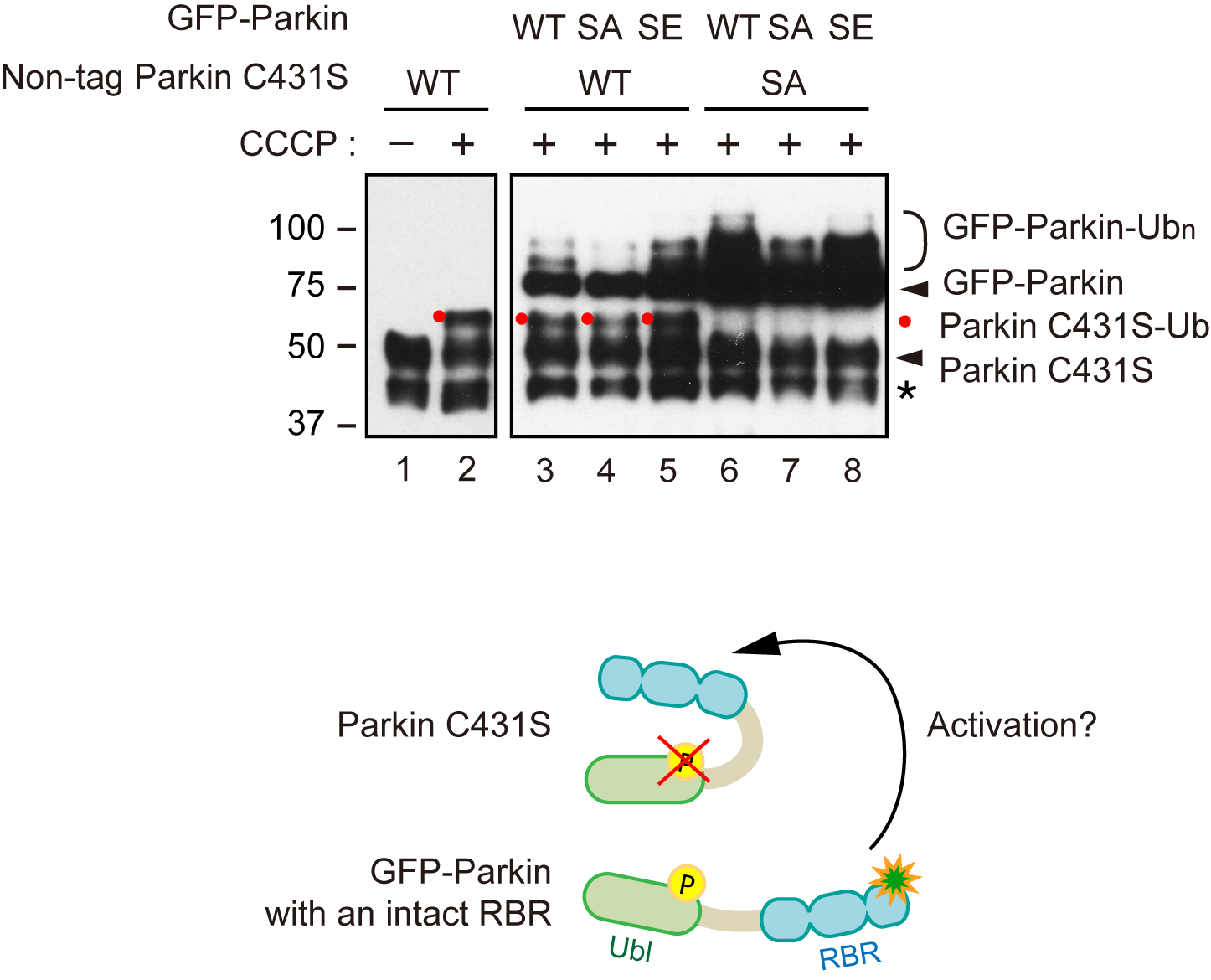

Supplement: Figure S5 — WT Parkin fails to activate the E3 activity of SA Parkin. HeLa cells were transfected with GFP-tagged human Parkin and untagged human Parkin C431S, in which Ser65 of the Parkin Ubl domain was replaced with Ala (SA) or Glu (SE), or intact (WT) as indicated. The cells were then treated with or without 30 µM CCCP for 3 h. Parkin C431S–ubiquitin oxyester formation (Parkin C431S-Ub) was monitored by western blotting with anti-Parkin. GFP-Parkin-Ubn, poly-ubiquitinated GFP-Parkin; asterisk, a processed form of Parkin. (TIF) [file pgen.1004391.s005.tif]
